# Active centromere and chromosome identification in fixed cell lines

**DOI:** 10.1186/s13039-016-0236-x

**Published:** 2016-03-22

**Authors:** Thian T. Beh, Ruth N. MacKinnon, Paul Kalitsis

**Affiliations:** Murdoch Childrens Research Institute, Royal Children’s Hospital, Parkville, Melbourne, VIC 3052 Australia; Department of Paediatrics, University of Melbourne, Royal Children’s Hospital, Parkville, Melbourne, VIC 3052 Australia; Victorian Cancer Cytogenetics Service, St Vincent’s Hospital, Fitzroy, Melbourne, VIC 3065 Australia; Department of Medicine, St Vincent’s Hospital, University of Melbourne, Fitzroy, Melbourne, VIC 3065 Australia

**Keywords:** Centromere, CENP-A, CENP-C, Immunofluorescence, Fluorescence *in situ* hybridisation (FISH), Multicolour FISH (mFISH), Neocentromere, Dicentric, Dicentric chromosome assay (DCA), Human erythroleukaemia (HEL) cell line

## Abstract

**Background:**

The centromere plays a crucial role in ensuring the fidelity of chromosome segregation during cell divisions. However, in cancer and constitutional disorders, the presence of more than one active centromere on a chromosome may be a contributing factor to chromosome instability and could also have predictive value in disease progression, making the detection of properly functioning centromeres important. Thus far, antibodies that are widely used for functional centromere detection mainly work on freshly harvested cells whereas most cytogenetic samples are stored long-term in methanol-acetic acid fixative. Hence, we aimed to identify antibodies that would recognise active centromere antigens on methanol-acetic acid fixed cells.

**Results:**

A panel of active centromere protein antibodies was tested and we found that a rabbit monoclonal antibody against human CENP-C recognises the active centromeres of cells fixed in methanol-acetic acid. We then tested and compared combinations of established methods namely centromere fluorescence *in situ* hybridisation (cenFISH), centromere protein immunofluorescence (CENP-IF) and multicolour FISH (mFISH), and showed the usefulness of CENP-IF together with cenFISH followed by mFISH (CENP-IF-cenFISH-mFISH) with the aforementioned anti-CENP-C antibody. We further demonstrated the utility of our method in two cancer cell lines with high proportion of centromere defects namely neocentromere and functional dicentric.

**Conclusions:**

We propose the incorporation of the CENP-IF-cenFISH-mFISH method using a commercially available rabbit monoclonal anti-CENP-C into established methods such as dicentric chromosome assay (DCA), prenatal karyotype screening in addition to constitutional and cancer karyotyping. This method will provide a more accurate assessment of centromere abnormality status in chromosome instability disorders.

## Background

One of the hallmarks of cancer is genome instability, often characterised by the presence of aneuploidy and genetic heterogeneity resulting from chromosome missegregation or defective DNA repair followed by the failure to enter cellular arrest or death [1, 2]. Such genetic heterogeneity ranges from the extent seen in leukaemias, generally presented with simple chromosomal rearrangements, to carcinomas that are often complex [3, 4]. It is only with cumulative method improvements and technological advancements made over the past 60 years that we are able to better understand disease mechanisms, and then apply the knowledge to cancer diagnosis, classification, prognosis, treatment selection and monitoring after treatment using the combination of molecular pathology, molecular cytogenetics and genomics in cancer research [5, 6].

One such technical advancement is the combination of RNA or DNA fluorescence *in situ* hybridisation (FISH) with immunofluorescence, commonly used for detection of RNA or DNA together with the protein of interest in or on the same cell. Co-detection of both genetic and the protein (epigenetic) components is especially crucial in determining the activity status of a centromere — whether it is functional (active) or non-functional (inactive). The human centromere is a DNA-protein structure consisting of the repetitive α-satellite DNA wrapping around nucleosomes containing CENP-A that specify the inner kinetochore onto which other kinetochore protein complexes assemble [7]. A properly functioning centromere is essential for correct chromosome segregation during cell divisions.

For cytogenetic investigations in the research setting, combinations of FISH and multicolour FISH (mFISH) performed on fixed cells as well as immunofluorescence followed by FISH (Imm-FISH) performed on freshly harvested cells are routinely used. However, for the studies of the centromere regions, most antibodies raised against human centromere proteins do not recognise the epitopes of their targets after fixation in methanol-acetic acid despite using the method proposed by Earnshaw et al. [8]. This includes immersing cell preparations into a low ionic strength buffer to unravel the conformation of the compact chromosome to improve accessibility of target antigen and then returning the cells to buffer at physiological ionic strength to restore the chromosome morphology [8]. As for the current Imm-FISH carried out on freshly harvested cells, the morphology of the chromosomes is often distorted due to the involvement of the cytocentrifugation step [9, 10].

In this paper, we report on and discuss (1) the screening outcome of several kinetochore antibodies for fixed cells, (2) the difference between the proposed method involving immunocytochemistry, FISH and mFISH, and the combination of other methods, and (3) the potential utility of the proposed method with the positive antibody, rabbit monoclonal anti-CENP-C, in identifying chromosomes with structural centromere defects in clinical samples of patients with congenital diseases or cancer as exemplified using T-47D, a breast cancer cell line, and SN12C, a renal cancer cell line, from the NCI-60 cancer panel.

For the comparison of methods aforementioned, the human erythroleukaemia (HEL) cell line was used because MacKinnon et al. [11] have shown by FISH that HEL has two large, rearranged chromosomes positive for multiple nucleolar organiser regions and three rearranged chromosomes that contain centromere DNA sequences from two different chromosomes. HEL has been widely used for cell biology and differentiation studies in addition to the extensive data generated from a variety of techniques namely whole chromosome painting, single nucleotide polymorphism (SNP) array, OncoMap sequencing, mFISH, multicolour chromosome banding (M-BAND) and targeted FISH [11].

Autoimmune antibodies in the sera of scleroderma patients were known to react to intranuclear antigens of tissue sections but a subset were pinpointed to stain the centromere region after substantiation with mitotic cells by Moroi et al. [12]. In 1985, the anti-centromere antibodies (ACAs) from the sera of CREST (calcinosis, Raynaud’s phenomenon, esophageal dismotility, sclerodactyly, telangiectasia) variant of scleroderma patients led to the discovery of the first three centromere proteins namely CENP-A (17 kDa), CENP-B (80 kDa) and CENP-C (140 kDa), named from the lowest molecular weight to the highest [13].

Some ACAs had been shown to work on methanol-acetic acid fixed cells. However, they usually recognise multiple centromere proteins depending on the individual serum and are limited in supply since they are restricted to individual autoimmune patients. Hence, ACAs are limited in their use for functional centromere identification where CENP-A and CENP-C are found exclusively on active centromeres but CENP-B is localised to the 17-bp CENP-B box of the centromeric repetitive DNA sequence regardless of its activity status [10, 14].

CENP-A and CENP-C are both part of the constitutive centromere-associated network (CCAN) that forms the inner kinetochore plate onto which other protein complexes assemble. CENP-A is a histone-H3 variant constituting the nucleosome core in a portion of the centromeric chromatin [15]. For CENP-A related studies, a mouse monoclonal antibody against human CENP-A (Clone: 3–19) generated by Ando et al. [16] has been widely used as it is known to give punctate signals that mark the inner kinetochore of the centromere region. Nonetheless, the binding of this antibody to CENP-A is obliterated if cells are fixed in methanol-acetic acid solution [16]. On the other hand, a rabbit polyclonal serum generated against full-length human CENP-C by the Earnshaw laboratory was shown to work on methanol-acetic acid fixed cells as reported by Warburton et al. [9]. In addition, CENP-C perfectly co-localises with CENP-A and both are constitutive markers of active centromeres [17] but the supply of the rabbit serum against CENP-C is limited.

## Results and discussion

### Centromere antibody screening

With the awareness that most samples in the cytogenetic laboratories are stored long-term in methanol-acetic acid fixative and with the expectation that centromere status screening will provide useful information for these laboratories, we decided to screen several commercially available antibodies that target components of an active centromere using fixed HCT-15, a near diploid and lowly rearranged human colon cancer cell line [4].

**Fig. 1:**
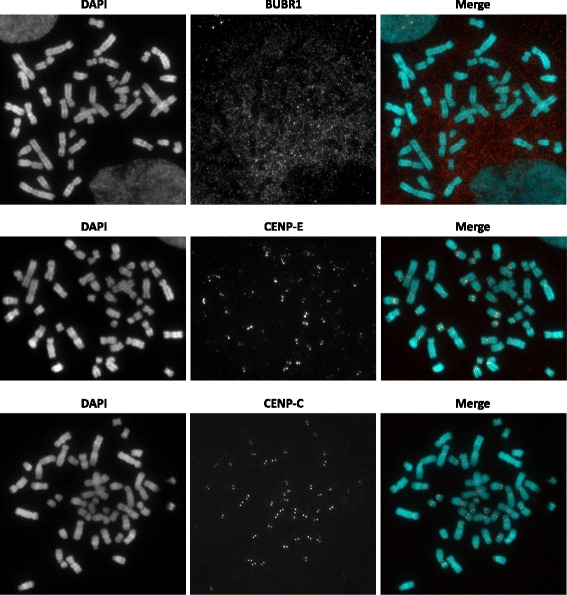
Active centromere antibody screening on methanol-acetic acid fixed cells. Representative images of immunocytochemistry using TEEN and KB buffer performed on HCT-15 cells stored in methanol-acetic acid fixative. Immunofluorescence images showing BUBR1, CENP-E and CENP-C in red and DAPI in cyan

From our screen, as summarised in Table 1, antibodies that did not show centromere signals on methanol-acetic acid fixed cells are the rabbit polyclonal antibody recognizing phosphorylated Ser18 of CENP-A (Active Motif) and the rabbit monoclonal against BubR1 [EPR12259(2)] (Abcam), a spindle assembly checkpoint protein of the *BUB1B* gene that plays a role in sensing proper chromosome-microtubule attachments during prometaphase to metaphase when it localises to the kinetochore (Fig. 1) [18]. The mouse monoclonal antibody against HEC1 (NDC80) [9G3] (Abcam), a protein of the NDC80 complex that stabilises microtubule-kinetochore binding, was also negative for centromere signals on fixed cells. Rabbit monoclonal against CENP-E [EPR4542(2)] (Abcam), a kinesin-like motor protein that accumulates at the kinetochore throughout metaphase [19], gave positive signals at the centromere regions but its staining was not homogeneous across all human chromosomes (Fig. 1), making it not ideal for the utilisation we were aiming for. Rabbit monoclonal against CENP-C [EPR15939] (Abcam) probed on fixed HCT-15 showed positive punctate signals (Fig. 1) similar to the signals seen in immunocytochemistry on non-fixed, cytocentrifuged cells even when it was used at a high dilution factor of 1 in 3,000 (data not shown).

**Table 1:** List of antibodies targeting components of active centromere. Antibodies were first tested on non-fixative treated cells to determine optimal concentration or dilution for immunocytochemistry and then tested on cells stored in methanol-acetic acid fixative

	Antibody	Species	Concentration or dilution	Cells stored in methanol-acetic acid
1	BubR1 [EPR12259(2)] ab183496 (Abcam)	Rabbit IgG monoclonal	0.527 μg/ml	No
2	CENP-A (Clone: 3–19)	Mouse IgG monoclonal	1 : 500	No
3	CENP-A phospho Ser18 (Active Motif)	Rabbit IgG polyclonal	1 : 400	No
4	CENP-C (Serum from rabbit 554; gift from William Earnshaw)	Rabbit polyclonal	1 : 1000	Yes
5	CENP-C [EPR15939] ab193666 (Abcam)	Rabbit IgG monoclonal	0.677 μg/ml	Yes
6	CENP-E [EPR4542(2)] ab133583 (Abcam)	Rabbit IgG monoclonal	0.275 μg/ml	Not homogeneous across all chromosomes
7	HEC1 [9G3] ab3613 (Abcam)	Mouse IgG monoclonal	0.5 μg/ml	No

### Combining cenFISH, CENP-IF and mFISH methods

**Fig. 2:**
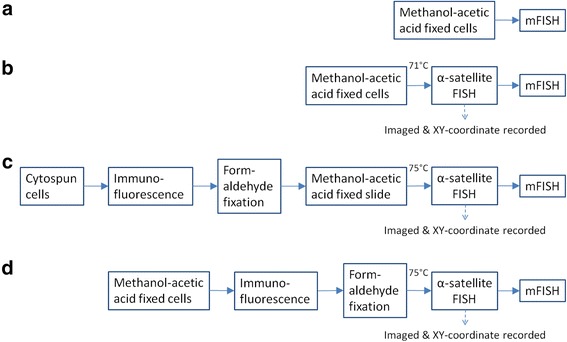
Workflow combining and comparing mFISH, cenFISH and CENP-IF. **a** Standard mFISH as suggested by MetaSystems. **b** cenFISH-mFISH: pan-centromeric FISH with pTRA7 α-satellite probes, designated as cenFISH, imaged with metaphase spreads coordinates recorded and underwent mFISH. **c** Freshly harvested cells swelled in hypotonic solution were cytospun onto microscope slide before immunofluorescence was performed using KCM buffer, cross-linked with 4 % formaldehyde, fixed and denatured with methanol-acetic acid, aged, underwent cenFISH and subsequently mFISH. **d** Immunofluorescence using TEEN and KB buffer was performed on cells stored in methanol-acetic acid fixative, cross-linked with 4 % formaldehyde before cenFISH and then mFISH was performed. Both methods **c** and **d** are CENP-IF-cenFISH-mFISH

**Fig. 3:**
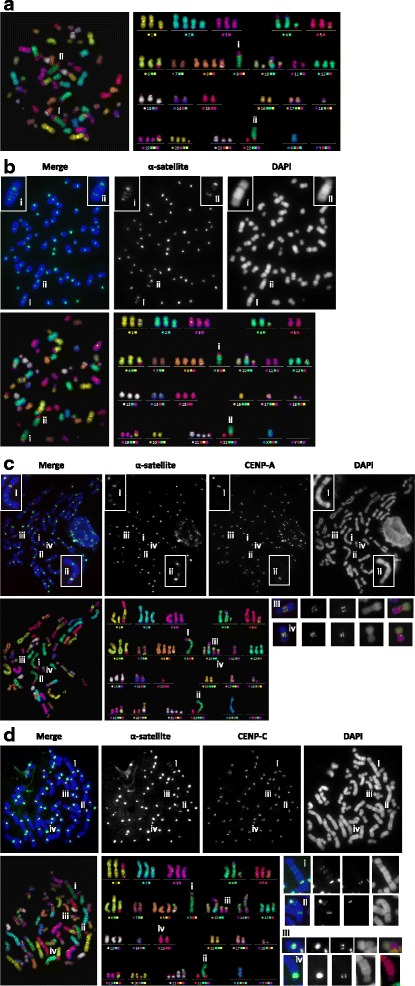
Representative images of HEL metaphase for each method, with marked (i) der(9) and (ii) psu dic(22;9). **a** Standard mFISH. Metaphase spread (*left*) and karyotype (*right*) in false colour. **b** cenFISH-mFISH. Upper panel: immunofluorescence images showing α-satellite (*green*) and DAPI (*blue*); insets are enlarged images of (i) der(9) and (ii) psu dic(22;9). Lower panel: metaphase spread and karyotype in false colour. **c** CENP-IF-cenFISH-mFISH with CENP-A. Upper panel: immunofluorescence images of cytospun cells showing α-satellite (*green*), CENP-A (*red*) and DAPI (*blue*); insets are enlarged images of (i) der(9) and (ii) psu dic(22;9). Lower panel: metaphase spread and karyotype in false colour display. (iii) and (iv) are der(10;19) with two pairs of CENP-A signals. **d** CENP-IF-cenFISH-mFISH with CENP-C. Upper panel: immunofluorescence images showing α-satellite (*green*), CENP-C (*red*) and DAPI (*blue*). Lower panel: metaphase spread and karyotype in false colour display; (i) and (ii) are der(9) and psu dic(22;9) respectively while (iii) is der(10;19) and (iv) is der(20)t(11;15;20) (as named in MacKinnon et al. [11]) observed with two pairs of CENP-C signals

After validating that the rabbit monoclonal against human CENP-C worked on fixed cells, we then cross-linked the primary and secondary antibodies before performing α-satellite FISH (cenFISH). This combined method of centromere protein immunofluorescence (CENP-IF) followed by cenFISH will be referred to as CENP-IF-cenFISH here onwards. In addition to the epigenetics and DNA-based information of the centromere activity, we believe that knowing the identity of the chromosomes would further add value to cytogenetic analysis. Hence, we attempted to carry out mFISH on the same cell spread on which CENP-IF-cenFISH had been performed prior and to ensure that mFISH would work sufficiently well post-CENP-IF-cenFISH, we compared the combination of methods as outlined in Fig. 2 using the HEL cell line (Fig. 3).

In general, the chromosome morphology of cytocentrifuged cells was not well-maintained compared to the metaphase spread of cells stored in methanol-acetic acid fixative (unpublished observation). However, we were still able to distinguish the chromosome identities with the mFISH procedure followed by image analysis (Fig. 3c) as seen in the comparable karyotypes generated from all methods (Fig. 3).

**Fig. 4:**
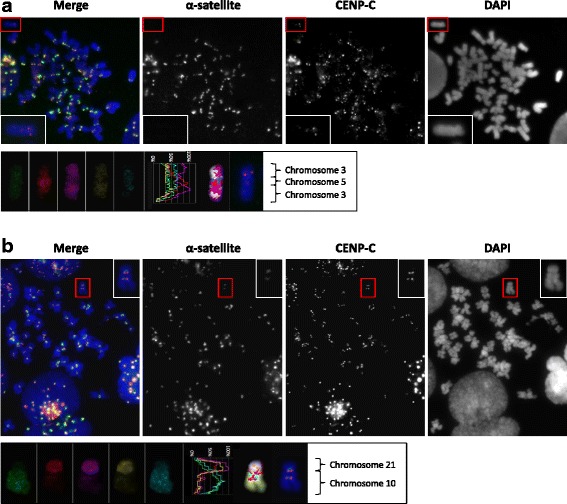
CENP-IF-cenFISH-mFISH performed on cancer cell lines T-47D and SN12C with neocentric and dicentric chromosome respectively. **a** Upper panel: immunofluorescence images for T-47D showing α-satellite (*green*), CENP-C (*red*) and DAPI (*blue*); boxed in red is the neocentric chromosome and insets are enlarged images of the neocentric chromosome. Lower panel: mFISH colour profile of the neocentric chromosome indicating it is a rearranged chromosome containing segments from chromosomes 3 and 5. **b** Upper panel: immunofluorescence images for SN12C showing α-satellite (*green*), CENP-C (*red*) and DAPI (*blue*); boxed in red is the dicentric chromosome and insets are enlarged images of the dicentric chromosome. Lower panel: mFISH colour profile of the dicentric chromosome indicating it is a rearranged chromosome from chromosomes 21 and 10

In our experiments, the cross-linking step with formaldehyde for antibodies against CENP-A and CENP-C prior to cenFISH in methods C and D respectively caused the red fluorescent signals at the active centromere regions to carry over into the mFISH analysis (Fig. 4b, lower panel). The green fluorescent signals from cenFISH were not detectable. Thus, by taking the carrying over of fluorescent antibody signal into account, we suggest the preparation of an additional sample but using a differently coloured secondary antibody against anti-CENP-A or anti-CENP-C for more accurate chromosome identification by mFISH post- CENP-IF-cenFISH if necessary.

### Comparison between CENP-IF-cenFISH-mFISH with CENP-A and CENP-C

Referring to Fig. 2, method A is the standard mFISH performed according to the protocol recommended by the manufacturer (MetaSystems) and it is a useful method to identify interchromosomal translocations but is unable to identify rearrangements involving α-satellite DNA especially the ones without observable constrictions. However, with method B, cenFISH followed by mFISH, multiple α-satellite signals were observed on two large chromosomes designated (i) and (ii) (Fig. 3b). Even in combination with DAPI staining of the DNA where chromosome constrictions may be identified, identification of the centromere was proven to be not trivial in HEL cell line especially on the chromosome denoted (i). However, through methods C and D (both are CENP-IF-cenFISH-mFISH), the presence of CENP-A and CENP-C signals indicates which of the two α-satellite positive constrictions was the active centromere [Fig. 3c (i) and 3D (i)].

As reported in MacKinnon et al. [11], three derivative chromosomes, namely der(4;20)t(4;11;20) and another two that resulted from whole arm translocations between two chromosomes, der(5;17) and der(10;19), contain centromere sequences originated from two different chromosomes. We were able to detect the presence of two active centromeres based on CENP-C signals on at least one der(10;19) chromosome in 2 out of 6 metaphase spreads with method C [Fig. 3c (iii) and (iv)] and in 4 out of 7 metaphase spreads using method D [Fig. 3d (iii)]. Der(5;17) and der(4;20) chromosomes were always showing only a pair of punctate CENP-A and CENP-C signals. Besides, the der(20)t(11;15;20) [Fig. 3d (iv)] in the metaphase spread of Fig. 3d was the only der(20) that showed 2 pairs of signals for CENP-C out of 7 metaphases analysed. This raised the possibility of the centromere of chromosome 15 being present on der(20) which could not be tested by MacKinnon et al. [11].

### Detection of neocentric and functional dicentric chromosomes

To demonstrate the method’s utility, method D which used anti-CENP-C was carried out on two cell lines, T-47D and SN12C. T-47D is a breast cancer cell line known to us for having a stable neocentric chromosome while SN12C is a renal cancer cell line that shows a high proportion of dicentric chromosomes in the metaphase spreads that we analysed for another study (in preparation). In both instances, our method was able to first detect centromere abnormalities within the metaphase spreads via CENP-IF-cenFISH (Fig. 4a & b, upper panels), followed by identification of the chromosomes that were involved in the final rearranged and centromere defect-bearing chromosomes (Fig. 4a & b, lower panels). The neocentromere in T-47D was found to be on a segment of chromosome 3 and the neocentric chromosome was a product of rearrangement between chromosome 3 and 5 (Fig. 4a, lower panel) whereas the dicentric chromosome in SN12C was formed from the rearrangement between chromosome 10 and 21 (Fig. 4b, lower panel).

In our study, we have demonstrated that our method, CENP-IF-cenFISH-mFISH, was able to (1) identify rearrangements implicating the centromeric DNA, (2) identify active centromere based on the presence of the constitutive kinetochore protein, CENP-C, and (3) reveal the identity of the chromosomes involved in the rearrangements for methanol-acetic acid fixed cells.

We hereby propose that this method will be particularly helpful in studying clinicopathologically complex groups of tumours, for example liposarcomas, potentially as an additional criterion in subcategorising them. Both atypical lipoma/well-differentiated liposarcoma (ALP-WDLPS) and dedifferentiated liposarcoma (DDLPS) are identified by the presence of similar marker chromosomes, namely the supernumerary ring or giant rod chromosome containing the amplified 12q14-15 region with amplification of the *MDM2* and *CDK4* genes. However, ALP-WDLPS is classified as having intermediate aggressiveness compared to DDLPS which is malignant [20]. In addition, a high proportion of ALP-WDLPS has a marker chromosome with a neocentromere in contrast to DDLPS that also has the marker chromosome containing amplified 12q14-15 but with alphoid-centromere [21, 22].

Functionally dicentric chromosomes with two active centromeres have been thought to be involved in the breakage-fusion-bridge cycle and have recently been suggested to also contribute to chromothripsis based on the modelling of a subset of pa ediatric acute lymphoblastic leukaemia (ALL) with intrachromosomal amplification of chromosome 21 [23]. Furthermore, in refining the treatment for patients of pa ediatric ALL, the presence or absence of a dicentric chromosome was considered alongside other cytogenetic and genomic criteria [24], and in a recent acute myeloid leukaemia (AML) study, the difference in median survival between patients with one dicentric (5.8 months) and those with three dicentric chromosomes (1.8 months) was shown to be significant [25]. Taken together, our method which has the capability to detect functional dicentric chromosomes in methanol-acetic acid fixed cytogenetic preparations could assist in understanding the involvement of dicentric chromosomes in disease mechanism and also in risk stratification of patients for the treatment of diseases other than childhood ALL and AML.

Furthermore, this method could also be applied to prenatal and congenital cases with chromosomal rearrangements containing centromere abnormalities that are not detected with current genomic technologies such as SNP microarrays and massively parallel sequencing [26]. An example of such chromosomal rearrangements is the isodicentric Y [idic(Y)], commonly found in children with disorders of sex development. Patients presented with idic(Y) are often mosaic with 45,X cells and a high proportion of females presented with 45,X Turner syndrome have the X chromosome of maternal origin, which together suggest that idic(Y) is mitotically unstable [27, 28]. In addition, idic(Y) is the most common structural anomaly of chromosome Y in infertile men exhibiting an abnormal Y chromosome [28]. An azoospermic prospective father with idic(Y) may seek assisted reproductive technologies (ART) to achieve parenthood but he risks transmitting the idic(Y) to his offspring. Hence, the detection of idic(Y) is important for pre- and postnatal genetic counselling as well as for genetic screening and recommendation of ART for infertile men. However, for routine screening, anti-CENP-C CENP-IF used together with chromosome specific FISH probe would be a more economical approach.

Another important application of the method is in the refinement of dicentric chromosome assay (DCA), the gold standard for biodosimetry assessment of individuals after exposure to radiation [29]. The International Atomic Energy Agency recommends an analysis of more than 1000 cells for better ascertainment of dicentric chromosome formation for low radiation dose of less than 100 mSv, equivalent to a computed tomography (CT) scan [30, 31]. A recent study by Abe et al. [31] reported that the analysis of 1000 metaphase spreads probed with cenFISH was sufficient to yield comparable precision to 2000 conventional Giemsa stained metaphases. However, these DCA methods only assess for the occurrence of a dicentric chromosome based on the presence of primary constrictions and centromere DNA, therefore, not providing any information on whether the affected chromosome contains one or two active centromeres which may have impacts on downstream genome instability. This gap of information could be addressed with the truncated version of our method D (excluding the mFISH step) as a DCA. With an additional 2 h and 40 min approximately for immunocytochemistry on top of cenFISH, it will provide the information on both the centromere DNA and its associated active kinetochore protein CENP-C, further improving the precision of the current DCA methods.

## Conclusions

The CENP-IF-cenFISH method using pan-centromeric FISH probe and commercially available rabbit monoclonal anti-CENP-C to detect active centromere performed on methanol-acetic acid fixed cells is an improvement on the existing DCA methods and prenatal and congenital testings for chromosome structure anomalies such as isodicentric Y. Furthermore, the CENP-IF-cenFISH-mFISH method which additionally reveals chromosome identity could potentially add diagnostic value and increase our understanding of disease mechanism if it was to be incorporated into established procedures including constitutional and cancer cytogenetic tests.

## Methods

### Cell culture

Cell lines namely HEL, HCT-15, T-47D and SN12C were cultured in Roswell Park Memorial Institute (RPMI) 1640 with 10 % fetal bovine serum, 2 mM L-glutamine, penicillin and streptomycin with 5 % CO_2_ at 37 °C [32].

### Cell preparation & immunocytochemistry

For immunocytochemistry performed on freshly harvested cells, colcemid (KaryoMAX, Thermo Fisher Scientific) was first added to cell medium to a final concentration of 0.1 μg/ml for 1.5 h. Cells were harvested via mitotic shake-off and subjected to hypotonic treatment (0.075 M KCl) for 15 min. 1500 cells were used for each slide preparation. Cells cytospun onto the glass slides were washed thrice with cold KCM (120 mM KCl, 20 mM NaCl, 10 mM Tris–HCl pH 7.5, 0.5 mM EDTA, 0.1 % Triton X-100) for 5 min each time and first incubated with mouse anti-human CENP-A (1:500) then followed by Alexa594 conjugated secondary antibody against mouse (1:1000) (Thermo Fisher Scientific), both diluted in KCM buffer with 1 % BSA, at 37 °C for 1 h and 40 min respectively, with three 5 min KCM buffer washes after each incubation. Cells were then cross-linked in KCM containing 4 % v/v formaldehyde (Merck Millipore) for 10 min at room temperature (RT), washed twice with distilled water, briefly once and 5 min for another, and air-dried before being fixed in ice-cold methanol-acetic acid (methanol:acetic acid, 3:1 volume ratio) at 4 °C for 30 min, air-dried and left to age for at least 48 h at RT. Antibody screening was performed on HCT-15 cells with the primary antibodies diluted according to Table 1 followed by Alexa594 conjugated secondary antibodies (1:1000) (Thermo Fisher Scientific) against the respective species. The cells were visualised without being fixed with methanol-acetic acid.

For immunocytochemistry on methanol-acetic acid fixed cells, cells were dropped onto glass slides and dipped immediately into TEEN buffer (1 mM triethanolamine-HCl pH 8.5, 0.2 mM Na EDTA, 25 mM NaCl) once the fixative had dried. TEEN buffer was changed twice, after 3 min. Cells were blocked with 0.1 % Triton X-100 (Sigma-Aldrich) and 0.1 % BSA (Sigma-Aldrich) in TEEN at 37 °C for 15 min followed by incubation in rabbit anti-human CENP-C (1:3000) (Abcam) in TEEN at 37 °C for 1 h. Slides were washed with KB buffer (10 mM Tris–HCl pH 7.7, 150 mM NaCl, 0.1 % BSA) thrice, 4 min each time before incubating with Alexa594 conjugated secondary antibody against rabbit (1:1000) (Thermo Fisher Scientific) diluted in KB buffer at 37 °C for 40 min. Slides were then washed twice with KB for 4 min each time before being fixed in KB containing 4 % formaldehyde for 10 min at RT, washed twice with water, briefly once and 5 min for another, and then air-dried. For antibody screening, after the washing step post-secondary antibody incubation, slides were mounted with Vectashield antifade mounting medium (Vector Laboratories) added with DAPI before visualisation.

### Pan-centromeric probes

The pTRA-7 plasmid containing pan α-satellite DNA (previously described in [33]) was labelled with biotin using the dCTP analog conjugated with biotin (Thermo Fisher Scientific). 2 μg plasmid DNA, 0.2 mM dNTP mix (Promega), NEBuffer 2 (New England BioLabs Inc.), 0.1 mg/ml BSA, 160 mU DNaseI (New England BioLabs Inc.) and 20 U DNA Polymerase I (New England BioLabs Inc.) were mixed into a final reaction volume of 30 μl and incubated at 15 °C for 2.5 h for nick translation. The reaction was then inactivated at 75 °C for 20 min. DNA was precipitated overnight at −20 °C with 20 μg of salmon sperm DNA (Thermo Fisher Scientific), 0.1 volumes NaOAc and 2.5 volumes of 100 % ethanol. Precipitated DNA was spun down, washed with 70 % ethanol and subsequently air-dried. The DNA pellet was then resuspended in 40 μl hybridisation buffer (30 % formamide, 2 X SSC, 10 % dextran sulfate) before being denatured at 95 °C for 5 min and then placed on ice. This was a modification based on Roche’s Nick Translation Kit protocol.

### FISH

For cells that were to be probed directly, colcemid was added to the cell medium to a final concentration of 0.1 μg/ml for 1.5 h. Cells were trypsinised, spun down for 4 min and washed once with phosphate-buffered saline (PBS) before being subjected to hypotonic treatment in 0.075 M KCl at 37 °C for 15 min. Cells were then fixed by adding ice-cold methanol-acetic acid, spun down, resuspended with the fixative after discarding the supernatant and this process was repeated once and the pellet was resuspended in a final 200–800 μl of fixative to yield an optimal cell density for metaphase spread preparation. Fixed cells were dropped onto glass slides and aged for at least 48 h at RT before performing FISH. Biotinylated probes against α-satellites were co-denatured with DNA on the slides at 71 °C for 5 min and incubated in a humidified chamber at RT for 16–18 h. Slides were then (i) washed using 2 X saline-sodium citrate (SSC) buffer twice followed by 1 X SSC buffer thrice, each time at RT for 5 min, (ii) blocked with Tris-NaCl-Blocking (TNB) [0.1 M Tris-HCl pH 7.5, 150 mM NaCl, 0.5 % w/v Blocking Reagent (Roche)] buffer at 37 °C for 30 min, (iii) incubated with avidin conjugated with Alexa-488 (dilution 1:500) (Thermo Fisher Scientific), (iv) washed thrice with 4 X SSC with 0.05 % v/v Tween-20 at 37 °C for 5 min each time and (v) mounted with Vectashield antifade mounting medium containing DAPI.

For cells that had undergone immunocytochemistry with CENP-A, aged slides were co-denatured with α-satellites probes at 75 °C instead of 71 °C for 5 min and incubated in a humidified chamber at RT for 16–18 h. Subsequent steps were the same as aforementioned.

### mFISH

mFISH was carried out with 24 XCyte (MetaSystems) according to the manufacturer’s instructions and with omission of a few early steps for slides that had undergone FISH and Imm-FISH. Slides that had undergone FISH only and immunocytochemistry followed by FISH were washed in 1 X PBS at RT for 3 min and 2 X SSC at 70 °C for 30 min, allowed to cool to RT for about 20 min, washed in 0.1 x SSC at RT for 1 min, denatured in 0.07 M NaOH at RT for 1 min, washed in 0.1 X SSC followed by 2 X SSC at 4 °C for 1 min each wash and then sequentially dehydrated in 30, 50, 70 and 100 % ethanol at RT for 1 min each before being air dried. Denatured 24 XCyte mFISH probes were then put onto the slides and incubated in a humidified chamber at 37 °C for 2 days. Post-hybridisation slides were washed in 0.4 X SSC at 72 °C for 2 min, 2 X SSCT (2 X SSC containing 0.05 % v/v Tween-20) at RT for 0.5 min and rinsed briefly in water before being air dried and mounted with Vectashield antifade mounting medium containing DAPI for visualisation.

### Microscopy and analysis

All images were taken using a Zeiss Axio Imager.M1 microscope/AxioCam Mrm camera. All Imm-FISH images were captured and analyzed with Axio Vs40 vs4.6.1.0 software (Carl Zeiss) while mFISH images were taken and analyzed with Isis colour fluorescence and FISH imaging system (MetaSystems).

## Abbreviations

cenFISH: centromere fluorescence *in situ* hybridisation
CENP-IF: centromere protein immunofluorescence
Imm-FISH: immunofluorescence followed by FISH
mFISH: multicolour FISH
RT: room temperature
